# Using knowledge of, attitude toward, and daily preventive practices for COVID-19 to predict the level of post-traumatic stress and vaccine acceptance among adults in Hong Kong

**DOI:** 10.3389/fpsyg.2022.1103903

**Published:** 2022-12-22

**Authors:** Yuan Cao, Judy Yuen-man Siu, Kup-Sze Choi, Nick Cho-lik Ho, Kai Chun Wong, David H. K. Shum

**Affiliations:** ^1^Department of Rehabilitation Sciences, The Hong Kong Polytechnic University, Kowloon, Hong Kong SAR, China; ^2^Mental Health Research Centre, The Hong Kong Polytechnic University, Kowloon, Hong Kong SAR, China; ^3^Department of Applied Social Sciences, Faculty of Health and Social Sciences, The Hong Kong Polytechnic University, Kowloon, Hong Kong SAR, China; ^4^School of Nursing, Faculty of Health and Social Sciences, The Hong Kong Polytechnic University, Kowloon, Hong Kong SAR, China; ^5^Centre for Smart Health, Faculty of Health and Social Sciences, The Hong Kong Polytechnic University, Kowloon, Hong Kong SAR, China

**Keywords:** COVID-19, KAP, knowledge – attitude – behavior, vaccine, PTSD

## Abstract

**Introduction:**

COVID-19 has been perceived as an event triggering a new type of post-traumatic stress (PTSD) that can live during and after the pandemic itself. However, it remains unclear whether such PTSD is partly related to people’s knowledge of, attitude toward and daily behavioral practices (KAP) for COVID-19.

**Methods:**

Through a telephone survey, we collected responses from 3,011 adult Hong Kong residents. Then using the Catboost machine learning method, we examined whether KAP predicted the participant’s PTSD level, vaccine acceptance and participation in voluntary testing.

**Results:**

Results suggested that having good preventative practices for, poor knowledge of, and negative attitude toward COVID-19 were associated with greater susceptibility to PTSD. Having a positive attitude and good compliance with preventative practices significantly predicted willingness to get vaccinated and participate in voluntary testing. Good knowledge of COVID-19 predicted engagement in testing but showed little association with vaccine acceptance.

**Discussion:**

To maintain good mental health and ongoing vaccine acceptance, it is important to foster people’s sense of trust and belief in health professionals’ and government’s ability to control COVID-19, in addition to strengthening people’s knowledge of and compliance with preventative measures.

## 1. Introduction

Given the long-lasting impacts of the pandemic, mental health problems also deserve attention. COVID-19 has been perceived as a new type of traumatic stress with serious mental health impacts, including PTSD-like responses (Bridgland et al., 2021; Kira et al., 2021). Feeling distant from people, sleep issues, difficulty concentrating, and intrusive thoughts have been reported as the most common symptoms of PTSD associated with the COVID-19 pandemic (Speth et al., 2020). Direct or indirect exposure to COVID-19, or even anticipation of such exposure events, can induce PTSD-like symptoms (Bridgland et al., 2021). Cognitive model of PTSS also proposed that PTSD occurs if a person processes a traumatic event with a feeling of the presence of a serious threat (Ehlers and Clark, 2000). In Hong Kong, although the prevalence of PTSD decreased from 28.6% in 2021 (Lau et al., 2021) to 12.4% in 2022 (Cao et al., 2022), its context-dependent nature means that its prevalence could increase again, as the risk of contracting COVID-19 changes with time (Sun et al., 2021). Thus, there is an urgent need to develop strategies to prevent and address the possible deterioration of the public’s mental health status.

Furthermore, COVID-19 vaccination and testing are crucial preventative measures in the context of relaxed social distancing rules (Aldila et al., 2021). In Hong Kong, despite a satisfactory level of vaccine acceptance, a decreasing trend in willingness to vaccinate has been reported in the literature (Wang et al., 2021). Participation in the “Universal Community Testing Program” implemented by the Hong Kong government in September, 2020 has not been high as well (Xin et al., 2022). Continuous attention to the uptake of these measures is essential.

Assessing people’s knowledge, attitudes, and practices (KAPs) not only helps health care professionals to provide appropriate assistance to individuals but also helps different sectors of society to establish a comprehensive plan to improve public health. KAP surveys have been used to investigate knowledge gaps and behavioral patterns related to effective health interventions (Papagiannis et al., 2020). The surveys can also be used to improve public health awareness campaigns and national disease control programs (Espinoza-Gómez et al., 2002). In this study, knowledge refers to the level of accurate knowledge about COVID-19. Attitude represents people’s thoughts, feelings, or beliefs about COVID-19 management. Practice refers to the preventive measures that the public has followed (Haq et al., 2012). The following sections illustrate how KAP relates to vaccine acceptance, participation in COVID-19 testing, and PTSD.

### 1.1. KAP and PTSD

Most studies on the relationship between KAP and mental health have focused on the impacts of the early stages of the pandemic. A high level of COVID-19-related knowledge was the greatest anti-PTSD protective factor among female college students, who were vulnerable to PTSD during the COVID-19 pandemic (Si et al., 2021). Nie et al. (2021) found that pandemic-related knowledge significantly predicted public panic, which in turn affected the incidence of PTSD.

Apart from people’s understanding of COVID-19, participants’ perception of the risk of infection, belief about the extent and emergency of the pandemic, and fear about the future were positively associated with the incidence of PTSD (Si et al., 2021). The perceived risk was also significant during the early stage when the lack of controllability of the pandemic was reflected in relevant information (Shi and Hu, 2004). Moreover, people with greater compliance with preventive measures recommended by the government and health care professionals, such as staying at home longer to ensure social distancing, may have negative psychological consequences, including post-traumatic stress (Ikizer et al., 2021). This association might be attributable to a lack of social support to cope with the pandemic and to stressful and traumatic perception of the pandemic (Ikizer et al., 2021).

Researchers have begun exploring the relationships among KAP, level of post-traumatic stress, and vaccine acceptance. However, most studies on COVID-19-related KAP have focused on the beginning stages of the pandemic. Research is needed to further investigate the ability of KAP to predict people’s mental health based on their preventative behaviors, and identify the critical predictors in an ongoing pandemic. This study focused on examining whether KAP predicted people’s level of psychological distress 1 year after the start of the pandemic in Hong Kong, and whether these factors affected their decision to get vaccinated and undergo testing.

### 1.2. KAP and vaccine acceptance

COVID-19-related KAP shape how people understand, think, and behave in relation to vaccination. Reluctance or refusal to get vaccinated was related to inadequate knowledge of COVID-19, particularly of the mode of SARS-CoV-2 transmission (Luk et al., 2021). For attitude toward COVID-19, people with greater perceived susceptibility (i.e., the subjective assessment of the risk of contracting SARS-CoV-2 infection) and more confident toward local health authorities in managing the spread of the virus had a higher tendency to get vaccinated (Chia et al., 2021; Yu et al., 2021). However, people with lower perceived severity and perceived threat of COVID-19 were unwilling to get vaccinated (Chia et al., 2021; Luk et al., 2021). As for preventative behaviors, Xiao et al. found greater compliance with social distancing measures among unvaccinated participants than among vaccinated participants (Xiao et al., 2022). Despite the growing compliance with preventive measures, and due to concerns about vaccine safety, a decrease in the willingness to receive COVID-19 vaccines was found (Wang et al., 2021).

### 1.3. KAP and participation in voluntary testing

COVID-19-related KAP have slightly different impacts on the willingness to undergo COVID-19 testing. A lack of knowledge and insufficient understanding of COVID-19 have been found to be associated with a lower participation rate in voluntary testing. People had limited understanding of the testing criteria, testing access, and test-qualifying symptoms (i.e., fever, cough, and loss of smell; Bevan et al., 2021; Graham et al., 2021). When the symptoms were mild, improved, or perceived as indicative of a flu instead of COVID-19, people did not undergo COVID-19 testing (Smith et al., 2021; Sudre et al., 2021). On the other hand, people with a higher perceived risk of infection, greater perceived severity of COVID-19, and greater concerns and negative emotions were more motivated to participate in voluntary testing (Fallucchi et al., 2021; Yue et al., 2021; Xin et al., 2022). Trust in the government’s control measures and the efficacy of voluntary testing may also have positively affected the participation rate in universal community testing programs (Xin et al., 2022). In addition, people who generally abide by the government’s preventive measures were more willing to undergo COVID-19 testing (Fallucchi et al., 2021; Thunström et al., 2021).

### 1.4. Hypothesis

Based on the results of the reviewed studies, we made the following hypotheses.

- KAP would be associated with the level of PTSD, vaccine acceptance, and participation in voluntary testing.
- Good knowledge of COVID-19, a trusting attitude toward the controllability of COVID-19, and less compliance with preventative practices would contribute to the willingness to receive vaccines and participate in voluntary testing.
- Poor knowledge, a pessimistic attitude, and good compliance with preventive practices would be associated with higher PTSD scores.

## 2. Materials and methods

### 2.1. Sampling method

Data collection was carried out from December 2020 to February 2021 by a contracting company that specializes in conducting telephone surveys. Random phone numbers were first generated using the common local prefixes, which were obtained from the Office of the Communications Authority. Half of the calls were made to landlines, while the other half to mobile phones. The participants were first given information about the survey, and verbal informed consent was obtained. The participants were then screened to confirm their eligibility to participate in the survey according to the following criteria. Inclusion criteria: (i) Cantonese-speaking residents of Hong Kong and (ii) aged 18 years or above. If the call was made to a household landline where there were multiple eligible respondents, the person whose birthday was closest to the date of call was chosen as the respondent from that household. Exclusion criteria: a minor below the age of 18, unable to speak Cantonese, or is not a Hong Kong resident.

### 2.2. Participants

The telephone survey was completed by 3,011 participants, including 1,596 females (53%). Most of the respondents were middle-aged or older adults (16% were 18–29 years old, 53.2% were 30–59 years old, and 30.8% were 60 years old and above). Most of the participants (81.1%) had an educational attainment of high school or above. In terms of employment status, most were employed full-time (45.7%), followed by retired (20.9%), and homemakers (10.7%). Most (60.4%) of the respondents were married. We believe that the sample size of 3,011 would provide reliable and accurate findings, with a 2% margin of error (population size of 6,413,800, 95% confidence).

### 2.3. Survey content

The data used in this study are part of a large-scale survey study on COVID-19. The complete survey contained six sections: (1) traumatic symptoms; (2) knowledge of, attitude toward, and preventative practices for COVID-19; (3) vaccine acceptance; (4) voluntary testing; (5) media exposure, and (6) demographic questions. This study examined whether KAP predicts traumatic symptoms and attitude toward vaccine acceptance and voluntary testing. The effects of demographic variables and media exposure on traumatic symptoms, attitude toward vaccine acceptance and voluntary testing, and behavioral practices are reported elsewhere (Cao et al., 2022).

To evaluate KAP, we adapted the three-part Questionnaire of Knowledge, Attitudes, and Practice Toward COVID-19 developed by Zhong et al. (2020), specifically for use in Hong Kong. The KAP approach has been used and validated previously in a Chinese sample (Zhong et al., 2020). The Knowledge section included 13 items examining the participant’s level of understanding of COVID-19. The response options were true, false, and do not know. A sample item was “People infected with COVID-19 are not contagious when they have no fever.” The Attitude section included two items asking the participant’s whether they thought that the pandemic will be controlled and if the spread of the virus will be stopped in Hong Kong. The response options were true, false, and do not know. The Practice section included 14 items asking participants to indicate the frequency at which they adopted the preventative measures recommended by the local health authority. The response options were always, often, sometimes, never, and not applicable/cannot answer.

To this questionnaire, we added two questions asking about vaccine acceptance and participation in voluntary testing: (i) “*Are you willing to receive a COVID-19 vaccine that is approved by the Department of Health?*” The response options were yes, no, and unsure; and (ii) “*Have you participated in the universal and free virus test conducted by the Department of Health in 2020?*”

The Chinese version of the Impact of Event Scale – Revised (CIES-R), validated by Wu and Chan (2003), was used to measure symptoms of possible PTSD. For this study, we adapted the context of the questions that were related to COVID-19 specifically. The Cronbach’s alphas for the Intrusion, Avoidance, and Hyperarousal subscales were 0.86, 0.82, and 0.79, respectively, in this study. The total score was used in the data analysis.

### 2.4. Data analysis

This study examined whether KAP can predict (i) the PTSD level, (ii) vaccine acceptance, and (iii) participation in voluntary testing. To this end, machine learning was adopted instead of statistical models due to its predictive accuracy. In particular, the categorial boosting algorithm Catboost was used for its distinctive ability to handle non-numeric categorical values with minimal transformation, which was instrumental for processing the data in this study. Catboost (Prokhorenkova et al., 2018) is a powerful decision tree-based ensemble machine learning method. It utilizes a greedy algorithm to combine categorical features at each split of a decision tree to produce increasingly effective features. As a supervised learning method, Catboost uses samples of input features and the corresponding known outputs to train a predictive model. In this study, the responses to the 29 items of the Questionnaire of Knowledge, Attitudes, and Practice Toward COVID-19 (Zhong et al., 2020) were adopted as inputs for the models. The models performed two-class classification, and the predicted outputs were binary. The construction of the models is detailed in the following paragraphs.

For the first scenario, the model, denoted as Model 1, was trained with Catboost to predict a high PTSD level based on whether the total score of the CIES-R (Wu and Chan, 2003) was greater than or equal to 33. The predicted PTSD level was normal if the total score was less than 33. In total, 2,632 samples fell into this scenario, with 322 positives (CIES-R score ≥ 33) and 2,310 negatives (CIES-R score < 33) at a ratio of approximately 1:7. The imbalance was counteracted using the technique of cost-sensitive learning (Thai-Nghe et al., 2010) whereby the minority instances (positives) were weighted more heavily in the training process to avoid biasing toward the majority class (negatives).

For the second scenario, the model predicted vaccine acceptance based on the responses to the survey question “Would you get a dose of vaccine that is approved by the Department of Health?” The question had four possible choices: “Yes,” “No,” “Do not know/Hard to tell,” and “Decline to answer.” Two models were built to predict vaccine acceptance, one predicting the outcome of either “Yes” or “No” and the other predicting either “Yes” or “Not-Yes,” where “Not-Yes” corresponded to the selection of one of the three non-Yes choices. These two models were denoted as Models 2 and 3, respectively. For the Yes-versus-No prediction in Model 2, there were 2,260 samples with 1,356 positives and 904 negatives (at a ratio of 1.5:1), and for the Yes-versus-Not-Yes prediction in Model 3, there were 2,978 samples with 1,356 positives and 1,622 negatives (at a ratio of 1:1.2). Cost-sensitive learning was applied in Model 2.

For the third scenario, the model, denoted as Model 4, was trained to predict the outcome of participation or non-participation in voluntary testing accordingly to the response to the survey question “Did you join the free COVID testing campaign held by the Department of Health in September 2020?” A total of 3,010 samples fell into this scenario, with 1,681 positives, and 1,329 negatives (a ratio of approximately 1.3:1).

In other words, we took a binary response (“yes” or “no”) from the questionnaire as input (e.g., the binary response to the item “I am confident that Hong Kong can successfully control COVID-19.”), and examined the likelihood of participants saying “yes” relative to those saying “no” with respect to the level of PTSD symptoms or vaccine acceptance (high or low), and reported these results in terms of odds ratios and *p* values.

In summary, the survey data provided pairs of inputs (the responses to the 29 items of the KAP questionnaire) and outputs (the CEIS-R score for Model 1, or responses to the corresponding survey items for Models 2, 3, and 4) for building models using Catboost. The samples and prediction outputs of the models are summarized in Table 1. The models were trained by 10-fold cross-validation repeated five times. The classification performance of the models was evaluated with six metrics, namely, area under the receiver operating characteristic curve (AUC), accuracy (ACC), average precision (AP), sensitivity (SEN), specificity (SPE), and F1 score (F1). Furthermore, among the 29 input features, the five that were most important for the prediction were identified during model training with Catboost, based on the feature importance values (FIVs), which represented the average change in prediction caused by changes in individual feature values. FIVs were normalized such that the sum of the FIVs of all of the input features was 100. The larger the FIV, the higher the importance. For the important input features identified, i.e., responses to survey items, the odds ratio corresponding to two groups of responses to each question, along with the value of *p*, were calculated.

**Table 1:** Prediction outputs and samples used to build the four prediction models.

Model	Prediction	Binary output	Sample size	Positive samples	Negative samples	Sample ratio
Model 1	PTSD level	High or Normal	2,632	332	3,210	1:7
Model 2	Vaccine acceptance	Yes or No	2,260	1,356	904	1.5:1
Model 3	Vaccine acceptance	Yes or Not-Yes	2,978	1,356	1,622	1:1.2
Model 4	Participation in voluntary testing	Yes or No	3,010	1,681	1,329	1.3:1

## 3. Results

**Figure 1:**
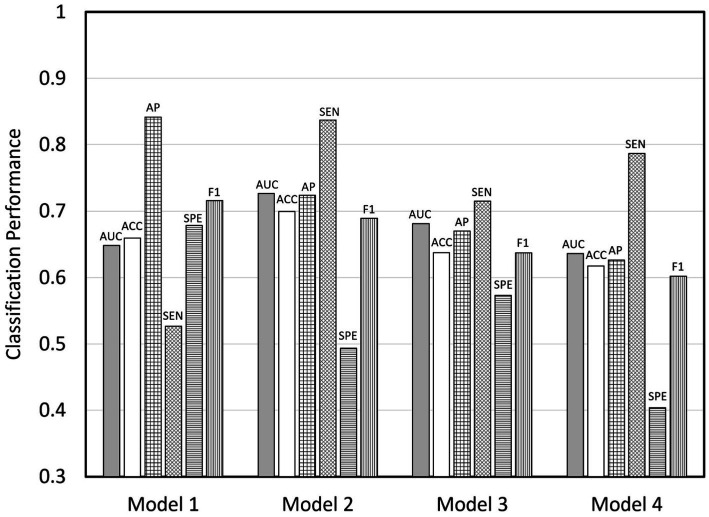
Bar charts showing the performance of the four prediction models. AUC, area under receiver operating characteristic curve; ACC, accuracy; AP, average precision; SEN, sensitivity, SPE, specificity; F1, F1 score.

The performance of the four prediction models, in terms of the means and standard deviations (*SDs*) of the six metrics, is tabulated in Table 2 and shown graphically in Figure 1. The AUC, ACC, and AP of the four models were all above 0.6, with Model 2 attaining the highest AUC and ACC at 0.7266 (*SD* = 0.0337) and 0.6995 (*SD* = 0.0300), respectively, and the second highest AP at 0.7238 (*SD* = 0.0364). The SEN of Model 2 was also the highest (mean = 0.8373, *SD* = 0.0313) among the four models. Overall, the performance of Model 2 appeared to be the best, although its SPE was below 0.5. In fact, the SPE values of all of the models were mediocre except for Model 1 whose SPE was close to 0.7. The SPE values of the rest of the models were below 0.6, with Model 4 exhibiting the lowest SPE at 0.4041 (*SD* = 0.0305). A comparison of Models 2 and 3, both of which examined vaccine acceptance, showed that Model 2 outperformed Model 3 in all but one (SPE) of the six metrics.

**Table 2:** Mean and *SD* (inside brackets) of six performance metrics of the prediction models.

Model	AUC	ACC	AP	SEN	SPE	F1
Model 1	0.6484 (0.0487)	0.6596 (0.0324)	0.8413 (0.0211)	0.5270 (0.0886)	0.6783 (0.0356)	0.7160 (0.0255)
Model 2	0.7266 (0.0337)	0.6995 (0.0300)	0.7238 (0.0364)	0.8373 (0.0313)	0.4937 (0.0463)	0.6889 (0.0316)
Model 3	0.6813 (0.0251)	0.6375 (0.0270)	0.6699 (0.0251)	0.7150 (0.0359)	0.5730 (0.0384)	0.6370 (0.0271)
Model 4	0.6360 (0.0342)	0.6175 (0.0284)	0.6260 (0.0332)	0.7867 (0.0317)	0.4041 (0.0431)	0.6020 (0.0305)

AUC, area under receiver operating characteristic curve; ACC, accuracy; AP, average precision; SEN, sensitivity; SPE, specificity; F1, F1 score.

The important features of the four models are given in Table 3, and the five most important features (i.e., survey items) of each model are listed in Table 4. The odds ratios corresponding to the two groups of responses to each item are also given in Table 4. Among the 29 items of the KAP questionnaire, 10 items were identified as important features in the predictive modeling. Item B “*I am confident that Hong Kong can successfully control COVID-19*” was among the top five features in all of the four models, ranking first in three models (Models 2, 3, and 4) and second in one model (Model 1). Furthermore, the odds ratios for this item were greater than 2 in all models (*p* < 0.0001), The odds ratios were even greater than 6 and 4 in Models 2 and 3, respectively. This suggested that, confidence with the local control or management of the COVID-19 infections was a key protective factor against having PTSD symptoms, as well as a motivator for receiving the COVID-19 vaccination. The FIVs of Item B were close to 30 out of 100 in Models 2 and 3, 12.6 in Model 4, and only 8.8 in Model 1. The FIVs of the top five important features in Model 1 were between 5 and 10.

**Table 3:** Important features of the four models.

Item	Important features in predictive modeling	Models
A	Eating or touching wild animals can cause COVID-19.	1, 4
B	I am confident that Hong Kong can successfully control COVID-19.	1, 2, 3, 4
C	Clean hands before touching the mouth, nose, or eyes.	1
D	Reduce leaving home and social activities.	1, 4
E	Not all infected people are seriously ill.	1
F	Avoid unnecessary social gatherings or dining.	2, 3, 4
G	Work from home or adopt staggered work hours.	2, 3, 4
H	Pay attention to toilet hygiene.	2
I	Maintain proper functioning of drainage pipes.	2, 3
J	Maintain environmental hygiene, e.g., sufficient indoor ventilation.	3

**Table 4:** Top five features of each model.

Model	Five most important features (Survey items)	Feature importance value	Groups of responses	Odds ratio	Value of *p*
1	A: Eating or touching wild animals can cause COVID-19.	9.2	Yes vs. No	1.5476	0.0022
	B: I am confident that Hong Kong can successfully control COVID-19.	8.8	No vs. Yes	2.0528	<0.0001
	C: Clean hands before touching the mouth, nose, or eyes.	8.5	Usually or more vs. Seldom or less	2.2748	<0.0001
	D: Reduce leaving home and social activities.	6.3	Usually or more vs. Seldom or less	1.6911	0.0008
	E: Not all infected people are seriously ill.	5.8	No vs. Yes	2.3265	<0.0001
2	B: I am confident that Hong Kong can successfully control COVID-19.	30.7	Yes vs. No	6.2338	<0.0001
	F: Avoid unnecessary social gathering or dining.	8.0	Always vs. Not always	2.2667	<0.0001
	G: Work from home or adopt staggered work hours.	6.8	Never vs. Not never	1.1932	0.0813
	H: Pay attention to toilet hygiene.	4.2	Not never vs. Never	1.2543	0.2224
	I: Maintain proper functioning of drainage pipes.	4.2	Usually or more vs. Seldom or less	1.9248	<0.0001
3	B: I am confident that Hong Kong can successfully control COVID-19.	29.5	Yes vs. No	4.7255	<0.0001
	F: Avoid unnecessary social gathering or dining.	6.9	Always vs. Not always	1.8053	<0.0001
	I: Maintain proper functioning of drainage pipes.	4.9	Usually or more vs. Seldom or less	1.5922	<0.0001
	G: Work from home or adopt staggered work hours	4.4	Never vs. Not never	1.2167	0.0404
	J: Maintain environmental hygiene, e.g., sufficient indoor ventilation.	3.9	Usually or more vs. Seldom or less	2.097	<0.0001
4	B: I am confident that Hong Kong can successfully control COVID-19.	12.8	Yes vs. No	2.3230	<0.0001
	F: Avoid unnecessary social gathering or dining.	9.6	Always vs. Not always	1.8526	<0.0001
	K: Eating or touching wild animals can cause COVID-19.	6.6	No vs. Yes	1.2681	0.0053
	D: Reduce leaving home and social activities.	5.6	Always vs. Not always	1.6589	<0.0001
	G: Work from home or adopt staggered work hours.	5.1	Never vs. Not never	1.4611	0.0003

Item F “*Avoid unnecessary social gathering or dining*” and Item G “*Work from home or adopt staggered work hours*” were both identified as among the top five important features in Models 2, 3, and 4. Item F was ranked as the second most important feature in these three models, with the odds ratios ranging between 1.8 and 2.3 (*p* < 0.0001). In comparison, the odds ratios of Item G in these three models were all below 1.5, and had lower statistical significance (*p* = 0.0818, 0.0404, and 0.0003, respectively). Four of the top five important features were shared by Models 2 and 3 (the exceptions were Item H in Model 2 and Item J in Model 3), which indicated their resemblance. In addition, Models 2, 3, and 4 shared three of the top five important features. Items D and K, which were important features in Model 4, were not among the five most important features in Models 2 and 3.

## 4. Discussion

Four machine learning models were built, with responses to the items of the KAP questionnaire as inputs, to predict people’s PTSD levels, vaccine acceptance, and participation in voluntary testing in the context of the COVID-19 pandemic in Hong Kong 1 year after it started. Good compliance with preventive measures and poor knowledge of and pessimistic attitude toward COVID-19 were factors associated with greater susceptibility to PTSD. Having a positive attitude and good compliance with preventative practices significantly predicted willingness to get vaccinated and participate in voluntary testing. Good knowledge of COVID-19 predicted engagement in testing but showed little association with vaccine acceptance. In particular, a positive attitude toward the controllability of the pandemic was a protective factor against PTSD and a motivator for vaccine acceptance. In contrast, good preventative practices were found to be a risk factor for higher PTSD scores, while good knowledge was protective against higher PTSD scores but had limited effect on vaccine acceptance.

### 4.1. Vaccination

The classification performance of the models suggests that responses to the KAP questionnaire could to a certain extent predict vaccine acceptance. Furthermore, using KAP to predict the decision to getting vaccinated was particularly promising, given the good performance of Model 2. This model also had a high SEN (0.8373). Although it was a variant of Model 2, the classification performance of Model 3 was comparatively lower, because the ambiguity of the “Not-Yes” responses in Model 3 obscured the prediction of vaccination-related decision with KAP. This suggests that it was more difficult to predict vaccine hesitancy than to predict refusal to get vaccinated, based on KAP responses alone.

The finding suggests that a positive attitude toward COVID-19 management and good compliance with preventative measures were more important in predicting vaccine acceptance than knowledge of COVID-19. In Models 2 and 3, only attitude toward COVID-19 (Item B) and preventive practices (Items F, I, and J) affected vaccine acceptance; the fifth most important feature, knowledge of COVID-19, did not have a significant effect. The lack of association between knowledge and vaccine acceptance may be because the vast, disparate, and even contradictory information spread through various media platforms, word-of-mouth, and health professionals undermined social trust in information (Wong et al., 2021). People could also have had information overload, and thus had difficulty in understanding all of the information (Holton and Chyi, 2012). Therefore, the local authorities and health professionals should focus on fostering people’s trust and belief in their abilities and health advice to better manage this health crisis (Lindholt et al., 2021; Yu et al., 2021; Xiao et al., 2022).

We then examined the important features that predicted vaccine acceptance, participation in voluntary testing, and PTSD level. Item B “*I am confident that Hong Kong can successfully control COVID-19*” was identified as a critical predictor for the three dependent variables, particularly for vaccine acceptance in Models 2 and 3 (FIVs close to 30 and odds ratios greater than 4). This is in line with our hypothesis and suggests that the general public’s decision to get vaccinated was largely dependent on their confidence in the controllability of the spread of the disease locally, that is in local health professionals’ or government’s ability to manage the pandemic. Another important feature was Item F “*Avoid unnecessary social gathering or dining*,” which was found to predict vaccine acceptance and participation in voluntary testing (Models 2, 3, and 4). This association indicates that promotion of compliance with such social distancing policies may lead to greater willingness to get vaccinated or undergo testing. This finding is not in line with the literature, which has suggested that there is a greater tendency to refuse vaccines among people more compliant to preventative measures, as they may believe that the daily preventative measures adequately protect their health (Wang et al., 2021; Xiao et al., 2022). This discrepancy may be due to the timing of the studies: we collected data approximately 1 year after the pandemic started, whereas most studies have focused on the beginning stage of the pandemic. It is possible that, over time, people are recognizing the limitations of the daily preventative measures or are perceiving vaccines to be part of the regular preventative measures.

### 4.2. Voluntary testing

The identification of the top five important features in Model 4 revealed the significance of KAP in predicting participation in voluntary testing. In this model, the most important feature was “*I am confident that Hong Kong can successfully control COVID-19*” (Item B), suggesting that a positive attitude toward COVID-19 control was related to a higher likelihood of participating in voluntary testing. Among features related to knowledge, a better understanding of Item A “*Eating or touching wild animals can cause COVID-19*” was related to greater willingness to undergo testing. However, mixed results were found for features related to preventative practice. Both the higher compliance items, Item D “*Reduce leaving home and social activities*” and Item F “*Avoid unnecessary social gathering or dining*,” and the lower compliance item, Item G “*Work from home or adopt staggered work hours*” were related to higher participation in voluntary testing. It is possible that the response to Item G was related to the nature of the participant’s work, such that they could not work from home (e.g., catering business) or that their employer did not allow flexible working hours. The findings related to knowledge of COVID-19 were in line with previous studies showing that the accuracy of people’s understanding of COVID-19 was positively related to their willingness to undergo testing (Graham et al., 2021; Smith et al., 2021; Sudre et al., 2021).

### 4.3. Post-traumatic stress

In Model 1, knowledge (Items A and E), attitude (Item B), and practice (Item C and D) were all represented in the five most important features. The FIVs of the top three important features (Items A, B, and C) were close, between 8.5 and 9.2, implying that these three features were equally important in determining a person’s emotional distress related to COVID-19. In fact, there was no dominant feature, as the FIVs of all of the five important features were relatively close and below 10. Specifically, the people with a poor understanding of how the virus is spread (Item A “*Eating or touching wild animals can cause COVID-19*”) or the potential severity of the disease (Item E “*Not all infected people are seriously ill*”) were more vulnerable to PTSD. Furthermore, a negative response to Item B “*I am confident that Hong Kong can successfully control COVID-19*” was associated with higher levels of PTSD. A higher compliance with certain preventative measures such as “*Clean hands before touching the mouth, nose, or eyes* (Item C)” and “*Reduce leaving home and social activities* (Item D)” was also related to higher levels of PTSD. These findings are in line with our hypothesis that poor knowledge of, pessimistic attitude toward, and good compliance with preventive practices for COVID-19 would be related to higher PTSD scores. This combination of predictors suggests that, even if people are following health advice on disease prevention, if they are not sufficiently knowledgeable of the disease or have negative attitudes or doubts about the controllability of the disease locally, they can experience psychological distress relating to the disease. This is consistent with prior research. For example, Si et al. reported that knowledge of COVID-19 served as a protective factor against PTSD, and that negative attitudes toward COVID-19 may be related to concerns about the risk of infection, worldwide impacts, and severity of the disease, all of which were positively associated with PTSD (Si et al., 2021).

### 4.4. Strengths and limitations

This study fulfills the research gap of lack of literature examining the strongest predictors on COVID-19 preventions and mental health during the pandemic. The results do shed light on people’s behavioral choices and mental health situation at approximately 1 year after the start of the pandemic. The multidimensional data was efficiently handled and analyzed using machine learning model. Together with a large and diverse sample, the predictive accuracy of the models enabled us to provide stronger conclusions. For the limitations, as the pandemic situation changes across different waves, more data could be collected at different time points to increase the accuracy of the prediction models. Furthermore, more algorithms could be compared in future studies to achieve the best prediction performance. We also acknowledge our sample might not be representative of the whole population in Hong Kong, as we could not obtain responses of people without a landline/mobile, or refused to participate in the study. Opinions of young people below the age of 18 were not included in this study. Regarding the content of the questionnaire, the measurements for KAP and the PTSD symptoms were based on previous studies using different Chinese samples. However, we were the first to apply them to the COVID-19 context in the Hong Kong Chinese population. Further research may be needed to confirm the suitability of the scales in this context. While outsourcing the telephone survey to a company was cost effective, it might introduce error in the data collection process. Measures had been made to reduce bias by withholding the hypothesis of the project form the data collection company, i.e., the data collectors were blind to the hypothesis. Therefore, systematic bias or error that could interfere with the overall pattern of results was minimized.

### 4.5. Conclusion

In conclusion, the results suggested that vaccine acceptance, PTSD symptoms and engagement in COVID-19 testing were all partly explained by levels of knowledge level, attitude, and daily preventative practices in relation to the COVID-19 pandemic. Among the three factors, having an optimistic attitude about the local management of the pandemic was found to be the key protective factor for the prevention of PTSD symptoms, and it was also the key motivator for vaccine acceptance. Therefore, to maintain good mental health and acceptance of ongoing vaccine boosters, it is important to foster people’s sense of trust in the ability of the health professionals and the government in controlling COVID-19, in addition to strengthening their knowledge of and compliance with preventative measures. Given the limitations of the project, care should be taken in interpreting the results. Future longitudinal studies would be useful, to establish a causal relationship between KAP and mental health, both during and post- the COVID-19 pandemic.

## Data availability statement

The datasets presented in this article are not readily available due to participants’ confidentiality. Requests to access the datasets should be directed to DS, david.shum@polyu.edu.hk.

## Ethics statement

The studies involving human participants were reviewed and approved by the Human Subjects Ethics Sub-Committee of The Hong Kong Polytechnic University. Written informed consent for participation was not required for this study in accordance with the national legislation and the institutional requirements.

## Author contributions

DS, YC, and JS were responsible for the conceptualization, funding acquisition, methodology, and investigation. YC, KCW, K-SC, and NH drafted the original manuscript. K-SC and NH conducted the data analysis. All authors provided important contributions for the interpretation of findings and contributed to the final version of the manuscript, read, and approved the final manuscript.

## Funding

This project was funded by the Health and Medical Research Fund, the Food and Health Bureau, The Government of the Hong Kong Special Administrative Region (Ref. no.: COVID190217). DS was supported by the Yeung Tsang Wing Yee and Tsang Wing Hing Endowed Professorship in Neuropsychology from the Hong Kong Polytechnic University. YC was supported by The Hong Kong Polytechnic University’s Start-up Fund for RAPs under the Strategic Hiring Scheme (P0038412). The funders of this study had no role in study design, data collection, data analysis or interpretation of data, writing of this article, and the decision to submit it for publication.

## Conflict of interest

The authors declare that the research was conducted in the absence of any commercial or financial relationships that could be construed as a potential conflict of interest.

## Publisher’s note

All claims expressed in this article are solely those of the authors and do not necessarily represent those of their affiliated organizations, or those of the publisher, the editors and the reviewers. Any product that may be evaluated in this article, or claim that may be made by its manufacturer, is not guaranteed or endorsed by the publisher.
